# Supplementary figures and images for: Tamilokus mabinia, a new, anatomically divergent genus and species of wood-boring bivalve from the Philippines
Source: PeerJ. 2019 Feb 7;7:e6256. doi: 10.7717/peerj.6256 (PMC6368970; doi:10.7717/peerj.6256)

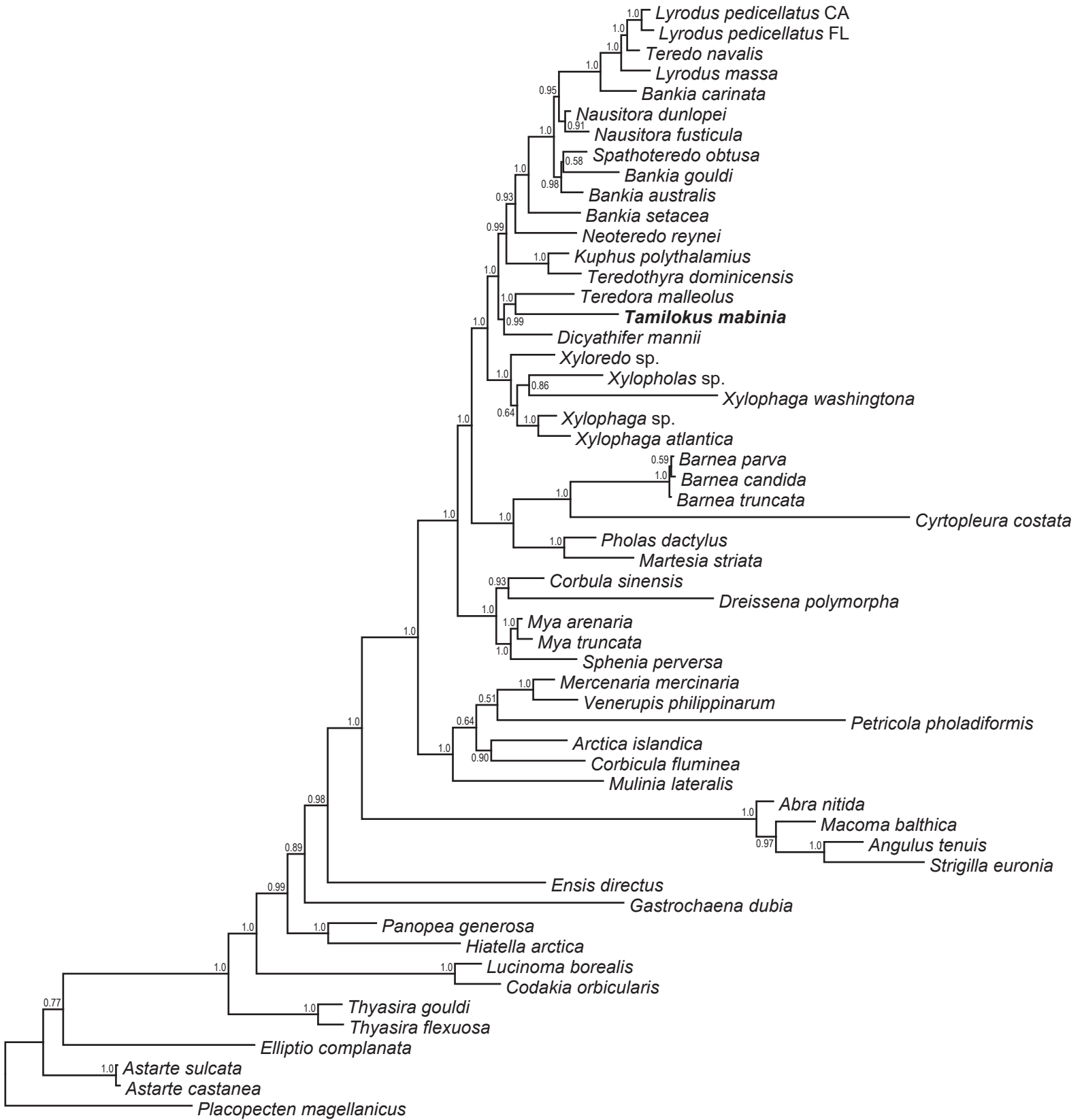

0.05

Supplement: Figure S1 — Bayesian analysis of the concatenated 18S and 28S nuclear rRNA gene sequences obtained from specimen PMS-3943P. The tree was constructed using the taxa presented in Distel et al. (2011). Numbers at nodes indicate posterior probabilities. Scale bars denote nucleotide substitutions per site. [file peerj-07-6256-s003.pdf]
